# Nutrition Label Reading and Understanding, Food Advertising Exposure, and Excess Weight Among Brazilian Adults: A Cross-Sectional Study

**DOI:** 10.3390/nu18040559

**Published:** 2026-02-08

**Authors:** Laysa Camila Bueno, Luiz Felipe de Paiva Lourenção, Thaiany Goulart de Souza-Silva, Cristina Garcia Lopes Alves, Marcelo Lacerda Rezende, Eric Batista Ferreira, Denismar Alves Nogueira, António Raposo, Zayed D. Alsharari, Mona N. BinMowyna, Sarah Almutairi, Daniela Braga Lima

**Affiliations:** 1 Faculty of Nutrition, Federal University of Alfenas, Alfenas 37130-001, MG, Brazil; laysa.bueno@sou.unifal-mg.edu.br (L.C.B.); luizfelipepaiva03@gmail.com (L.F.d.P.L.); cristina.lopes@unifal-mg.edu.br (C.G.L.A.); 2 Department of Morphology, Federal University of Minas Gerais, Belo Horizonte 31270-910, MG, Brazil; thaiany300@gmail.com; 3 Institute of Exact Sciences, Federal University of Alfenas, Alfenas 37130-001, MG, Brazil; marcelo.rezende@unifal-mg.edu.br (M.L.R.); eric.ferreira@unifal-mg.edu.br (E.B.F.); denismar.nogueira@unifal-mg.edu.br (D.A.N.); 4 CBIOS (Research Center for Biosciences and Health Technologies), ECTS (School of Health Sciences and Technologies), Lusófona University, Campo Grande 376, 1749-024 Lisboa, Portugal; 5 Department of Health Rehabilitation Sciences, Faculty of Applied Medical Sciences, University of Tabuk, P.O. Box 741, Tabuk 71491, Saudi Arabia; zalsharari@ut.edu.sa; 6 Applied College, Shaqra University, Shaqra 11911, Saudi Arabia; m.mwena@su.edu.sa; 7 Department of Community, Psychiatric, and Mental Health Nursing, College of Nursing, Qassim University, Buraydah 51452, Saudi Arabia; sh.almutairi@qu.edu.sa

**Keywords:** excess weight, obesity, nutrition labeling, food advertising, food choices

## Abstract

**Background/Objectives:** Nutrition labeling and food advertising are population-level strategies that may influence food choices. Excess weight is a recognized public health concern and a risk factor for cardiometabolic diseases; however, evidence regarding the association between label use, food advertising, and excess weight remains inconsistent. The objective of this study was to examine the associations between nutrition label reading and understanding, exposure to food advertising, food-related behaviors, and excess weight among Brazilian adults. **Methods:** A cross-sectional study was conducted with 580 adults living in the southern region of Minas Gerais, Brazil. Data were collected using a structured questionnaire addressing sociodemographic characteristics, food purchasing behaviors, exposure to food advertising, and habits related to reading and understanding nutrition labels. Excess weight was assessed using body mass index (BMI), calculated from self-reported weight and height. Logistic regression models and principal component analysis (PCA) were performed, adopting a significance level of 5%. **Results:** Excess weight was observed in 59.0% of participants. Regular use of nutrition labels was reported by 38.6% of respondents; among these individuals, 70.4% reported discontinuing the purchase of a food product after reading its nutritional information. In adjusted analyses, age over 30 years (*p* < 0.001), female sex (*p* = 0.006), higher number of dependents (*p* = 0.007), and type of media used (*p* = 0.005) were significantly associated with excess weight. The habit of reading nutrition labels was not independently associated with excess weight; however, better label understanding was associated with changes in food purchasing decisions. Considering the nutritional quality of foods as an important factor in food choices was associated with lower odds of having excess weight, although this association did not reach conventional levels of statistical significance (OR = 0.403; 95% CI: 0.15–1.00; *p* = 0.056). **Conclusions:** Excess weight among Brazilian adults was more strongly associated with sociodemographic and behavioral factors than with the habit of reading nutrition labels. Although nutrition labeling was not directly associated with excess weight, label understanding and perceived nutritional quality influenced food purchasing behaviors. These findings highlight the role of nutrition labeling and food advertising in shaping food choices and underscore the need for longitudinal studies to clarify their relationship with excess weight and related health outcomes.

## 1. Introduction

Cardiovascular diseases (CVDs) remain a leading global cause of mortality [1]. In Brazil, CVDs account for nearly 28% of annual deaths, mainly affecting the poorest people in the population and those with difficulty accessing health services [2]. Furthermore, between 2006 and 2019, the prevalence of diabetes and hypertension in the country increased from 5.5% to 7.4% and from 22.6% to 24.5%, respectively [3]. The development of CVDs is influenced by complex and multifactorial factors, including general obesity (or abdominal obesity), diabetes, and hypertension [4]. Treatment for both CVDs and obesity typically involves drug therapies, accompanied by lifestyle changes and modifications in eating behavior [5,6,7]. Notably, studies have shown that food and nutritional education aimed at weight management is associated with improvements in quality of life and indicators related to cardiometabolic health, particularly in populations with arterial hypertension [8,9]. A reduction in the body mass index (BMI) across the Brazilian population has the potential to reduce death rates from chronic non-communicable diseases (CNCDs) by approximately 25.3% with a potential annual prevention of 14.9% of all deaths [10].

Food and nutrition education has long been recognized as an important component of strategies aimed at reducing risk factors for cardiometabolic diseases, particularly in the context of obesity and related conditions. Evidence indicates that dietary patterns characterized by high consumption of ultra-processed foods, excess sodium, added sugars, and saturated fats substantially increase the risk of obesity, hypertension, and cardiovascular diseases. Although clinical nutrition counseling and cardiac rehabilitation programs play an important role in secondary prevention, their reach is limited at the population level. Therefore, public health strategies increasingly emphasize preventive approaches that promote food literacy, informed decision-making, and healthier dietary behaviors across diverse social and economic contexts [11,12].

In this context, nutrition labeling emerges as a population-based educational and regulatory strategy aimed at improving consumers’ understanding of food composition and supporting healthier food choices in everyday settings. Unlike individualized educational interventions, nutrition labeling operates at the structural level of the food environment, providing standardized information at the point of purchase and potentially influencing dietary behavior without the need for direct professional guidance. Previous studies have shown that the effectiveness of nutrition labels depends not only on their availability but also on consumers’ ability to interpret and use the information provided, highlighting the relevance of nutritional literacy and label comprehension as mediators between information exposure and health-related outcomes [13,14].

Nutritional labels are commonly utilized as a tool in food and nutritional education (FNE) to facilitate healthier dietary choices [15,16] and are recognized as a key strategy to inform food choices related to obesity and cardiometabolic risk factors. Remarkably, Matos et al. (2019) [17] demonstrated that among various determinants, such as age, education, income, and others, nearly 40% of the studied population does not habitually read nutritional labels on food products. Additionally, 33% of individuals exhibited limited proficiency in interpreting the information presented on these labels [17].

Given the increasing life expectancy of the Brazilian population [18], the rising prevalence of individuals with CNCDs [19], and the significance of nutritional labels as a potentially valuable tool in FNE [20], it is imperative to assess consumers’ knowledge of nutritional label information that allows monitoring and guiding the intake of specific nutrients, such as saturated fats, added sugars, and sodium; behaviors in relation to food labeling; and their association with obesity and broader health-related factors.

This article is relevant based on the analysis of the historical context of recent decades, observing the antagonism of temporal trends between malnutrition and obesity, characteristics brought about by the nutritional and demographic transition, with a strong tendency towards the consumption of processed and ultra-processed foods and a consequent increase in the consumption of saturated fatty acids, sugars, soft drinks, alcohol, industrialized products with excess “trans” fatty acids, meat, milk, and derivatives rich in fat, and sweets such as sweets, chocolates, and candies, among others [21].

Such food components, in addition to contributing significantly to the increase in overweight and obesity and CNCDs, underscore the need for a rigorous food regulatory agenda, from the perspective of nutritional knowledge, on the part of the population and the improvement of the right to information, in a clear way and precisely, with the aim of protecting consumers from potentially abusive and misleading practices and promoting food autonomy [22]. To examine the associations between nutrition label reading and understanding, exposure to food advertising, food-related behaviors, and excess weight among Brazilian adults.

## 2. Materials and Methods

### 2.1. Participants

The present study is a descriptive-exploratory, cross-sectional study with a quantitative approach, conducted in the South macro-region of the state of Minas Gerais, Brazil, which comprises 14 health micro-regions. The total population of the south macro-region is estimated to be 2,868,635 inhabitants, accounting for approximately 12% of the entire population of Minas Gerais [23]. For the sample calculation, the total resident population of the studied health macro-region was considered, aiming to configure a representative sample, and a confidence level of 95%, an error margin of 5%, and the known population size were considered, resulting in a recommended sample size of 385 participants. To account for a potential 50% increase in the minimum sample size, a total of 580 participants were selected between October 2020 and June 2021, during the COVID-19 pandemic period in Brazil, using the “virtual snowball” data collection method. This non-probabilistic data collection technique allows the definition of a sample through referrals made by participants, who indicate others that meet the research inclusion criteria [24]. It is worth noting that the selection probabilities are unknown; therefore, network sampling is non-probabilistic, making it impossible to calculate the natural weights of the sampling design. To obtain a representative sample of the population, the distribution of participants was observed according to geographic location and sociodemographic characteristics by municipalities and the total resident population of the studied health macro-region, seeking to create a representative sample.

$$n=\frac{{z^{2}}_{(1-\gamma )/2}\;p(1-p)}{d^{2}}$$

*n* = minimum sample size, *p* = proportion; *z*^2^(1 − *γ*)/2 = quantile of the standard normal distribution; and *d* = tolerable absolute error.

With the onset of the COVID-19 pandemic and the consequent adoption of measures restricting physical contact, health research with in-person interviews and data collections was limited. In addition to adhering to protective measures and restricting physical contact and taking into account the need to acquire knowledge about diseases and other public health problems, new research methods were encouraged, such as encouraging the use of the Internet, in order to obtain health information quickly and to assess the most diverse health issues [25,26].

To this end, research in a virtual environment is a current trend for data collection and has encouraged researchers to use online questionnaires as an alternative method for obtaining responses in scientific research, including health surveys. The chain sampling procedure, such as the methodology known as “snowball”, has been used and adapted to virtual social networks to collect information. Thus, the use of this methodology is justified, since the collection was carried out during the COVID-19 pandemic [27].

### 2.2. Variables

For this study, a structured questionnaire was used, adapted from instruments previously validated and applied in similar Brazilian populations [28,29]. The questionnaire was organized into thematic sections addressing: (i) sociodemographic characteristics; (ii) eating behaviors and food purchasing patterns; (iii) exposure to and influence of food advertising; and (iv) habits related to reading and interpreting nutrition labeling. Items related to food labeling and media influence were adapted from previously published surveys, with minor semantic adjustments to ensure clarity and suitability for the general adult population. As the original instruments were available in Portuguese, no translation or back-translation procedures were required.

Variables related to media were operationalized as indicators of exposure and use (e.g., type of media accessed for food-related information). The questionnaire did not assess trust in media sources, perceived credibility, or individual susceptibility to advertising. Therefore, media-related variables in this study should be interpreted strictly as measures of exposure rather than attitudinal or psychological constructs.

Due to the constraints imposed by the COVID-19 pandemic and the exclusive use of an online data collection strategy, no formal pilot testing of the questionnaire was conducted prior to data collection. This limitation is acknowledged and considered in the interpretation of the findings.

The questionnaire was administered as a single structured instrument, and no formal psychometric validation or internal consistency analysis was performed for the adapted version, which represents a limitation of the study. Table 2 presents selected variables derived from the questionnaire items related to the use and interpretation of nutrition labels. The complete questionnaire used for data collection is provided as Supplementary Materials to allow transparency and reproducibility.

The recruitment of participants was conducted virtually through telephone calls or popular social networking platforms (WhatsApp and Facebook, Meta Platforms Inc., Menlo Park, CA, USA). In the body of the message, in addition to presenting the research, there was a request for the participation invitation to be shared with the person’s network of contacts. Only individuals between the ages of 18 and 60, possessing undisputed autonomy, with a minimum educational attainment of elementary school, and residing in the municipalities comprising the south macro-region were included in the study. Exclusion criteria encompassed individuals under 18 or over 60 years old and those residing outside the south macro-region of Minas Gerais. Interested individuals who met the inclusion criteria and provided informed consent were given a questionnaire to complete.

All data collected in this study were self-reported. Anthropometric information, including body weight and height, was obtained through declarative measures and used to calculate body mass index (BMI), which was subsequently classified according to standard cut-off points. No clinical examinations, laboratory tests, or medical records were accessed. Therefore, all variables related to excess weight reflect self-reported information and should be interpreted considering the potential for reporting bias.

Participants were categorized into two groups based on their engagement with reading nutritional labels: those who regularly read nutritional labels and those who did not. BMI, calculated from self-reported weight and height, served as the dependent variable, while other independent variables, including economic, sociodemographic, and lifestyle factors, were analyzed separately for each group based on label reading. BMI was used as an indicator for the presence of obesity. No clinical examinations, laboratory tests, or cardiometabolic outcomes were assessed in this study.

### 2.3. Ethics Approval and Consent to Participate

The study was approved by the Research Ethics Committee of the Federal University of Alfenas (UNIFAL-MG) (CAAE: 33571520.1.0000.5142/Protocol No.: 4.209.111/2020), on 12 August 2020, in compliance with the principles of the Declaration of Helsinki.

### 2.4. Statistical Analysis

Data analysis was conducted using R statistical software, version 4.1.2 (R CORE TEAM, 2021). Normality of data distribution was analyzed using the Shapiro–Wilk test. Descriptive analysis was performed to estimate frequency distributions, means, and standard deviations for continuous variables. Inferential analysis included estimation of parameters of interest, presented as point estimates and by 95% confidence intervals. Categorical variables were analyzed applying *chi-square* independence tests, while comparisons of means for normally distributed variables were conducted using a *t-test*. To identify associated factors, a multiple logistic regression model was utilized, with a significance level set at 5%.

The selection of variables for inclusion in the multivariable logistic regression models followed an a priori theoretical approach, based on conceptual relevance and evidence from the literature. Multicollinearity among independent variables was assessed using variance inflation factors (VIFs), and no evidence of problematic collinearity was observed. Model adequacy was evaluated using goodness-of-fit criteria. Potential interaction terms, including sex × nutrition label reading, were tested; however, no statistically significant interactions were identified and therefore were not retained in the final models. Missing data were assessed for all variables included in the analyses. Participants with missing information on any of the variables of interest were excluded from the multivariable models. Therefore, all analyses were conducted using a complete-case approach.

Furthermore, a principal component analysis (PCA) was performed using the FactoMineR package (versão 2.4) to explore the interrelationships among variables [30].

## 3. Results

### 3.1. Characterization of the Population Under Study

The study sample consisted of 580 participants, with a mean age of 35.7 ± 10.67 years. The majority of participants were female (80.2%), had at least some undergraduate education (74.7%), reported family incomes below two minimum wages (37.9%), engaged in regular physical activity (57.6%), and were classified as having excess weight (59.0%). Among those diagnosed with excess weight, 37.4% were classified as overweight, while 21.6% were classified as obese (Table 1). According to secondary data from the SISVAN system, the prevalence of overweight and obesity among residents of the South macro-region of Minas Gerais was 68.9% among individuals monitored by primary health care. A higher frequency of overweight (38%) was observed in men compared to women (34%). However, obesity was more prevalent in women (35% in the study sample) compared to men (25%).

### 3.2. Habit, Use, Interpretation, and Influences of the Nutritional Labels

Food label use was reported by 38.6% of participants who habitually read food packaging. Among the interviewees, 51.0% expressed difficulty in comprehending the terms used in the labels, which was further assessed through specific questions such as “A product with reduced sodium content is …?” and “Can a product claiming to have a “low caloric value” be considered light?”. When examining the influence of labeling information, such as “reduced caloric value”, “light”, “diet”, “enriched”, and “source of vitamins”, 33.6% of consumers reported an average influence on their purchasing decisions, while 49.8% indicated either minimal or no influence. Notably, 70.4% of consumers reported discontinuing the purchase of a food product after reading its nutritional information, primarily due to concerns related to high calorie, sodium, fat, and/or carbohydrate content (Table 2).

**Figure 1:**
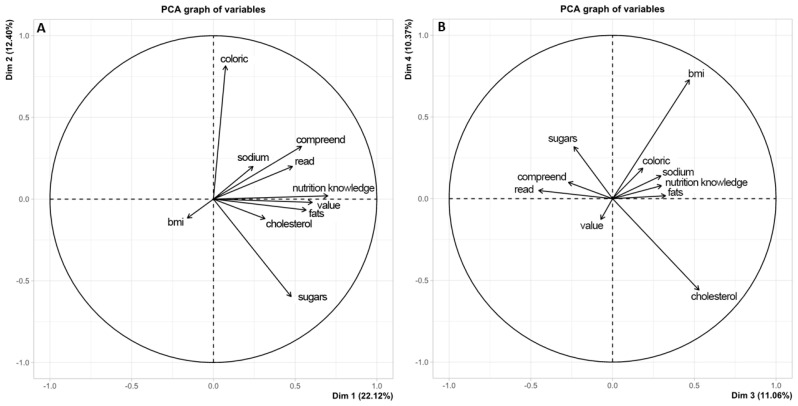
Principal component analysis assessing the relationship between understanding of food labeling and body mass index (BMI). (**A**) Main components chart illustrating the relationships between variables related to the understanding of food labels. (**B**) Projection of body mass index (BMI) in relation to label comprehension variables. Sodium—understanding of the claim “reduced sodium content”; Cholesterol—interpretation of the claim “cholesterol free”; Fats—understanding of the claim “reduced total fat content”; Caloric—interpretation of the claim “low caloric value” as light; Sugar—interpretation of the claim “low sugar content” as diet; Value—interpretation of the claim “reduced energy value”; Score—total score derived from questions related to food composition and labeling.

Investigating the relationship between understanding food labels and the prevalence of obesity, principal component analysis revealed that 55% of the total variance was explained. However, no significant correlation was found between the use and understanding of labels and the prevalence of obesity (Figure 1A).

Nevertheless, Figure 1B demonstrates a notable association between label usage and understanding, as depicted by the vectors representing “reading” and “understanding”. Furthermore, positive relationships were observed between the “score”, “value” and “fats” vectors. Participants were also queried about the factors influencing their food purchasing decisions, including price, nutritional quality, food flavor, brand, media (food advertising), practicality, and packaging appearance. Notably, practicality (27.1%), nutritional quality (37.1%), price (37.2%), and, predominantly, food flavor (57.6%) emerged as the key factors influencing the decision-making process. Media exerted the least influence among the variables (40.7%). The principal component analyses were conducted for exploratory purposes, aiming to visualize patterns of association among variables rather than to establish causal or inferential relationships.

### 3.3. Use and Influence of the Media

The Internet emerged as the primary communication medium utilized by the majority of study participants 90.0%, while 20.6% reported placing trust in other media sources. Participants typically spent 1 to 3 h per day engaging with these communication networks. Notably, 81% of the participants acknowledged feeling attracted to and subsequently purchasing products due to advertisements.

**Figure 2:**
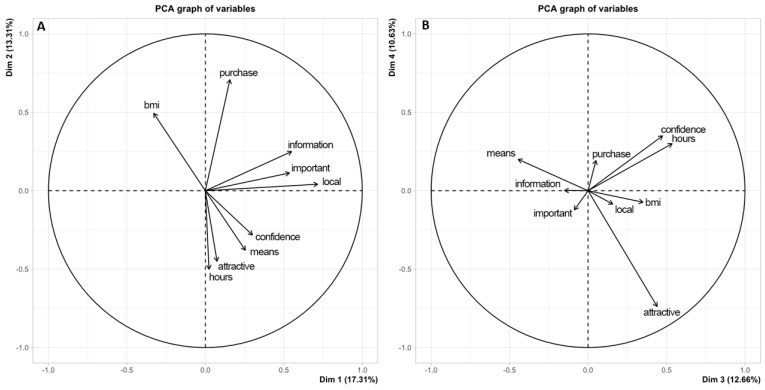
Principal component analysis assessing media use and body mass index (BMI). (**A**) Main components chart illustrating the relationships between variables related to media use and nutrition information. (**B**) Projection of body mass index (BMI) in relation to media use variables. Means—media most frequently followed; Importance—perceived importance of nutrition labeling; Information—use of labels to obtain nutritional information and support a healthy diet; Place—location on the package where nutritional quality information is presented; Attractive—attractiveness of food advertising influencing purchase; Purchase—person responsible for food purchasing in the household; Hours—daily time spent following media (including internet); Confidence—level of trust in the media usually followed; BMI—body mass index.

Figure 2A displays the principal components graph showing an inverse relationship between BMI and the use of the internet as a source of information, showing that the lower the BMI, the greater the use of the internet as a source of information, which may inform strategies aimed at controlling excess weight. In contrast, individuals with high BMI had a positive relationship with the use of other media (television, radio, magazines, among others). Furthermore, Figure 2B demonstrates a positive relationship between individuals who spent more time using communication networks and the highest degree of trust.

To explore the relationship between age and obesity, intriguing findings emerged. As age advanced, there was a notable increase in the number of individuals diagnosed with obesity. Specifically, individuals in the age range of 30–39 years exhibited approximately 3.1 times higher odds of being obese (CI: 2.02–4.97), while those aged 40–49 years had 3.2 times higher odds (CI: 1.88–5.63) and individuals aged 50–59 years had 3.5 times higher odds of being obese (CI: 1.93–6.72) (*p* ≤ 0.001). Additionally, intriguing patterns were observed regarding family composition, with individuals from households comprising more than three people demonstrating an increased likelihood of being overweight (OR = 1.779; CI: 1.17–2.73; *p* = 0.007) and utilizing services provided by Basic Health Units (OR = 2.007; CI: 1.22–3.33; *p* = 0.006). We also identified factors associated with lower odds of being overweight or obese within the population under investigation. Notably, individuals who primarily relied on the Internet as their main source of information demonstrated lower odds of being overweight or obese (OR = 0.388; 95% CI: 0.19–0.74). Moreover, being female was associated with reduced odds of being overweight or obese (OR = 0.515; CI: 0.32–0.82). Furthermore, considering the nutritional quality of food as a significant factor in food choices was associated with lower odds of having excess weight (OR = 0.403; 95% CI: 0.15–1.00; *p* = 0.056) (Table 3). Although consideration of nutritional quality in food choices was associated with lower odds of having excess weight, this association did not reach statistical significance at the conventional α = 0.05 threshold. Therefore, it should be interpreted as a suggestive trend rather than evidence of a protective effect.

The habit of reading nutrition labels was not independently associated with excess weight and did not remain in the final adjusted model (OR = 0.92; 95% CI: 0.71–1.18; *p* = 0.510).

## 4. Discussion

This cross-sectional study examined the associations between nutrition labeling, food advertising, eating behavior, and excess weight in a sample of 580 individuals aged 18 to 60 years. Our findings indicate that there is no significant association between nutrition labeling, food advertising, and BMI in the study population. However, we observed a positive association between the use of food labels and healthier food choices, as evidenced by 70.4% of the individuals refraining from purchasing certain food products after reading the nutritional information.

Although consideration of nutritional quality in food choices was associated with lower odds of having excess weight, this relationship did not reach statistical significance at the conventional α = 0.05 threshold. Therefore, this finding should be interpreted as a suggestive trend rather than evidence of a protective effect. Given the cross-sectional design of the study, reverse causation and residual confounding cannot be ruled out, as individuals with excess weight may be more likely to report greater concern about nutritional quality. Longitudinal studies are needed to clarify the direction and magnitude of this association.

Interestingly, our data indicate an inverse association between reported internet use and excess weight. However, this finding should be interpreted with caution, as the cross-sectional design precludes causal inference and does not allow conclusions regarding cardiometabolic health or disease prevention. These findings suggest a pattern of co-occurrence between nutritional label reading and food-related decision-making behaviors; however, recommendations for widespread adoption should be interpreted cautiously given the cross-sectional nature of the study. Such patterns may be addressed through educational campaigns and increased interaction between consumers and healthcare professionals. Aligning with the Dietary Guidelines for the Brazilian Population, food and nutritional education plays a pivotal role as a tool in developing individuals’ skills, given the significance of dietary choices in promoting healthier eating patterns and long-term cardiometabolic health [31,32].

Our findings revealed that only 38.6% of the participants reported regularly reading nutrition labels, highlighting a concerning trend consistent with previous studies conducted by Kye et al. (2020) and Nieto et al. (2020) [33,34]. These studies reported that less than 50% of the population examined actively engaged with nutritional labels when making food purchase decisions. Notably, research conducted in Mexico and South Korea suggested that individuals with obesity, diabetes, or a combination of CNCDs were less likely to use nutritional labels and exhibited lower levels of high-density lipoprotein (HDL-c) and higher triglyceride levels [33,34]. As the frequent consumption of food products rich in saturated fatty acids, trans fats, and simple carbohydrates constitutes risk factors for cardiovascular diseases [35], consulting nutritional labels may support informed food choices that are associated with lower intake of critical nutrients, although the impact on CNCD risk cannot be inferred from cross-sectional data. Additionally, a low-sodium diet can contribute to controlling systemic arterial pressure and to reducing the likelihood of cardiovascular events [35].

Regarding understanding of nutritional labels, our results indicated that 51% of the population exhibited limited comprehension of the available information. Interestingly, literature suggests the need to adapt the nutritional labeling model to a more accessible language for the entire Brazilian population, as acronyms and technical terminology pose challenges for understanding [20,32,36]. Therefore, it can be inferred that the inadequate nature of nutritional labels in terms of comprehension for the Brazilian population [20] may represent a contextual barrier to the adoption of healthier food choices at the population level. Individuals with greater knowledge about nutritional labeling tend to report more frequent use of this information when making food choices, a pattern that has been described in association with healthier dietary profiles in the literature [33]. It is important to emphasize that the principal component analysis (PCA) was conducted as an exploratory analytical approach aimed at identifying patterns of co-occurrence among the study variables. The groupings observed in the PCA plots represent descriptive associations based on shared variance and should not be interpreted as causal, directional, or hierarchical effects. Therefore, the PCA findings are intended to complement the regression analyses by illustrating underlying relational structures in the data rather than establishing explanatory or predictive mechanisms.

After recognizing the issue of a nutritional labeling standard that did not meet the needs of the Brazilian population, the National Health Surveillance Agency (*Agência Nacional de Vigilância Sanitária*, ANVISA) took action in 2020 by publishing RDC No. 429 and Normative Instruction No. 75, which introduced new technical requirements for food labeling [37]. These changes encompassed layout standardization and the inclusion of critical health-related information on ingredients or additives present in food products [36]. This strategic approach aligns with the Strategic Action Plan for Coping with CNCDs in Brazil, with a particular emphasis on enhancing label visibility and readability to facilitate consumer understanding [38,39].

It is important to situate the present findings within the Brazilian regulatory context of food labeling. Data collection for this study occurred prior to the full implementation of RDC No. 429/2020 and Normative Instruction No. 75/2020, which introduced mandatory front-of-package labeling and standardized warning symbols in Brazil. Therefore, participants were predominantly exposed to the previous labeling model, which may partly explain the limited comprehension of nutritional information and the weak associations observed between label use and excess weight. The effects of the new regulatory framework on consumer understanding and food-related behaviors warrant investigation in future studies conducted after its full implementation.

An additional finding of this study, although not statistically significant, suggested a tendency to associate individuals who choose low-nutritional-quality products with the ‘overweight’ variable. Studies conducted by Bonanni et al. (2013) and Veríssimo et al. (2019) [40,41] have demonstrated that the habit of reading labels directly impacts eating behavior, leading to reduced sugar consumption and increased intake of whole grains. Considering these findings, nutrition labeling may be understood as a relevant informational component within broader food environments, particularly among populations with a higher prevalence of excess weight, although its effectiveness and impact require confirmation in longitudinal and intervention studies [40,41].

Another influential factor in the purchasing decision is food flavor. Our study indicated that 57.6% of the study population considered food flavor as one of the determining factors when selecting food products. This finding aligns with the research conducted by Bialkova et al. (2016) [42], which revealed that consumers often associate a healthy diet with less palatable foods, leading them to repeatedly purchase based on their hedonic experience without considering the nutritional aspects [42]. Although it is important to dispel the misconception that healthy eating equates to bland flavor, we did not find an association between obesity and individuals who prioritize food flavor in their purchasing decisions.

The study identified an inverse association between BMI and reported internet use; however, this finding should be interpreted with caution, as causal direction cannot be established. Furthermore, our data demonstrated a significant association between using other media as information sources and excess weight (*p* = 0.005). It is important to emphasize that the present study evaluated media use and exposure, rather than trust in media content or individual susceptibility to advertising. These constructs, although related, represent distinct dimensions and were not directly assessed in this analysis.

Communication media play a crucial role in disseminating information on various topics, including eating habits and nutrition. Particularly, during the unprecedented period of the COVID-19 pandemic and the resulting social distancing measures, the percentage of individuals searching for health-related information on the Internet increased from 55% in 2019 to 72% in 2021, respectively [43,44]. However, the information available on social networks may not always be reliable which may partly explain the association between social media use and higher BMI. It is important to note that although virtual communities targeting individuals with cardiovascular diseases have shown positive effects on treatment, including improvements in blood pressure parameters, cardiovascular risk score, and physical activity levels, these benefits are not sustained over time [45,46]. Therefore, they cannot replace the importance of ongoing monitoring and care provided by qualified healthcare professionals.

This association is likely influenced by residual confounding factors, such as age, educational attainment, occupational profile (including remote work), and socioeconomic status, which were not fully captured in the present analysis. Therefore, this association should not be interpreted as causal but rather as a contextual indicator related to broader lifestyle and demographic patterns.

Regarding the impact of advertisements on purchasing decisions, our study revealed that 81% of participants reported buying food products influenced by advertising. Moreover, there is a growing trend in the industry to develop food products labeled as “diet”, “light”, “enriched with vitamins”, “high in fiber”, or “trans fat-free”. However, it is noteworthy that such technical terms are frequently used to attract consumers’ attention to ultra-processed and unhealthy food products [47,48].

When considering the age distribution in our study population, it is notable that individuals in the 30-year-old age range had the highest prevalence of overweight (above 60%). These findings support previous research indicating a correlation between aging and increased body weight [49]. Additionally, our data suggested lower odds of having excess weight among women in this sample with an estimate of obesity reduction, contrary to findings from the National Health Survey (*Pesquisa Nacional de Saúde*, PNS) and Cardiovascular Statistics studies [49,50]. This discrepancy could be attributed to other aspects associated with CVDs that were beyond the scope of this study, such as age above 65 years, serum cholesterol levels, smoking, and sedentary lifestyle [50].

In our study, we observed that 58% of the population analyzed exhibited excess weight (BMI ≥ 25 kg/m^2^). These findings are consistent with data from the PNS, which reported a 60.3% prevalence of overweight or obesity in the Brazilian population [44], thereby ratifying our outcomes. When examining different macro-regions, data from the 2019 SISVAN revealed that nearly 70% of the population in the south macro-region had a BMI above the ideal range, highlighting the association between excess weight and healthcare utilization, as demonstrated in our study [51,52].

Existing literature suggests a correlation between income and the purchase of higher-quality foods, characterized by increased consumption of fruits and vegetables, reduced consumption of ultra-processed products, and consequently, lower body weight [53,54]. Consistent with these findings, our study indicated that families consisting of four to eight individuals relying on a minimum wage showed a different likelihood of having a BMI ≥ 25 kg/m^2^ (*p* = 0.007). This association can be attributed to social inequalities and lower education attainment, which lead economically disadvantaged populations to consume more affordable, calorie-dense, and nutritionally poor foods. Therefore, socioeconomic variables alongside physical aspects contribute to the development of obesity [55,56]. Nutrition labeling is intended to provide consumers with information about food composition, potentially supporting healthier food choices. However, this does not mean that consumers are using it as a tool to choose which foods should comprise the diet and thereby reduce overeating and adverse health outcomes.

In the area of Public Health, the results of this study point to an important strategy to be adopted in conjunction with Public Health Promotion Policies, allowing the population to make healthier food choices, contributing to their knowledge and autonomy, and informing decisions and regulatory strategies in order to facilitate the understanding of nutritional information present on food labels. The same should be taken into consideration in relation to food advertising, via TV commercials and product placement, in view of its effect on eating behavior, and may inform public health strategies aimed at improving food-related decision-making [57].

From a practical perspective, the findings suggest that certain types of nutrition label claims were more frequently misunderstood by consumers, particularly those related to functional and health-related claims. Such misinterpretation may lead to overly favorable perceptions of ultra-processed or nutritionally imbalanced products. These results underscore the need for targeted consumer education strategies aimed at improving the interpretation of label claims and front-of-package information. In addition, clearer regulatory communication and stricter monitoring of health-related claims may help reduce consumer confusion and support more informed food choices.

### Strengths and Limitations of the Study

This study has several important limitations that should be acknowledged. First, its cross-sectional design precludes the establishment of temporal or causal relationships between the observed associations. In addition, anthropometric data were obtained through self-reported measures, which may have introduced classification errors and reporting bias.

Another important limitation relates to the sample composition and recruitment strategy. Participants were predominantly women and individuals with higher educational attainment, which likely reflects the use of online recruitment through social networking platforms. This sampling approach may have resulted in selection and coverage bias, particularly relevant for outcomes related to health literacy, nutrition label comprehension, and food choice behaviors. Consequently, the observed levels of nutrition label use and understanding may be overestimated, while difficulties faced by individuals with lower educational attainment and reduced access to digital resources may be underestimated. Moreover, the relatively young age profile of the sample may have influenced the observed associations, as younger adults tend to have greater exposure to digital information and potentially higher familiarity with nutrition labeling.

Furthermore, the restricted age range (18–60 years) limits the generalizability of the findings. Older adults, who bear a higher burden of cardiovascular disease, were not included; therefore, the results should not be interpreted as representative of the general adult population nor used to infer cardiovascular risk at the population level. Instead, the findings reflect behavioral patterns related to nutrition labeling and food choices within a specific subgroup of adults.

Data collection was conducted remotely during the COVID-19 pandemic, a period marked by substantial changes in daily routines, food purchasing patterns, lifestyle behaviors, and access to food. Social restrictions, economic instability, and increased reliance on digital food environments may have influenced eating behaviors, exposure to food advertising, physical activity habits, and body weight, potentially affecting the associations observed in this study. Although online data collection offers advantages such as broader geographic reach, lower cost, anonymity, and convenience, these benefits must be weighed against the potential for selection bias and limited representativeness. Therefore, the findings should be interpreted considering this exceptional context.

Despite these limitations, this study has notable strengths. It adopts a comprehensive approach by integrating behavioral, social, and demographic dimensions and includes a large sample from the southern region of Minas Gerais. The use of appropriate statistical methods, including logistic regression and principal component analysis, strengthens the consistency of the analyses. Moreover, the study has high public health relevance, as it contributes to understanding how nutrition labeling and food advertising influence food-related behaviors, offering insights to support food and nutrition education strategies and regulatory policies.

Considering these findings, future studies should explore the impact of nutrition labeling and food advertising in more diverse sociocultural and economic contexts. Longitudinal and intervention designs may help elucidate causal relationships between label reading and understanding and outcomes related to obesity and cardiometabolic risk.

## 5. Conclusions

This study provides valuable insights into the associations between nutrition labeling, reading habits, and exposure to food advertising on food purchasing decisions. Our findings suggest that understanding nutrition labels, rather than merely developing a habit of consulting them, is a more relevant factor influencing food choice. Accordingly, no significant association was observed between the habit of reading labels alone and the prevalence of excess weight or cardiometabolic risk factors.

Improving the accessibility and clarity of nutrition labels is crucial to support informed food choices across populations with diverse educational backgrounds. Labels should use clear, straightforward language and avoid overly technical terminology to facilitate comprehension. While more user-friendly labels may promote healthier dietary behaviors, these findings should be interpreted cautiously, as the cross-sectional design precludes causal inferences. Nonetheless, the study contributes to discussions on nutrition education, public health strategies, and regulatory policies, including the role of food advertising in shaping consumer behavior.

This research advances the literature in three key dimensions: (i) examining obesity patterns in relation to sociodemographic characteristics; (ii) exploring associations between nutrition labeling, food-related behaviors, obesity, and cardiometabolic risk; and (iii) situating these relationships within the context of a developing country undergoing nutritional and demographic transitions.

In conclusion, nutrition labeling and related informational factors are associated with food purchasing behaviors in the adult population studied. Although no direct link was observed between label reading and excess weight, label understanding and perceived nutritional quality were more strongly connected to healthier food choices. Given the study’s cross-sectional design and sample characteristics, causal relationships cannot be established. These findings underscore the relevance of nutrition labeling in shaping dietary behaviors and highlight the need for longitudinal and intervention studies to clarify its impact on obesity and cardiometabolic health.

## Figures and Tables

**Table 1:** Characteristics of the population under study.

Characteristic	N	%
**Gender**		
Female	465	80.2
Male	115	19.8
**Marital status**		
With a partner	373	64.3
Without a partner	207	35.7
**Schooling**		
≤High school	147	25.3
>High school	433	74.7
Family income *		
<2 wages	220	37.9
2–3 wages	143	24.7
3–6 wages	142	24.5
>6 wages	75	12.9
**Family size**		
≤3 people	320	55.2
>3 people	260	44.8
**Area of residence**		
Rural	49	8.5
Urban	531	91.5
**Use of public health services**		
Yes	497	85.7
No	83	14.3
**Nutritional status (BMI)**		
Low weight	11	1.9
Eutrophic	227	39.1
Overweight	217	37.4
Obesity	125	21.6
**Physical activity**		
Yes	334	57.6
No	246	42.4

* Minimum wage considered: BRL 1100.00.

**Table 2:** Use and interpretation of food labels.

Characteristic	N	%
**Do you read the food labels?**		
Yes	224	38.6
No	356	61.4
**Do you consider labeling important?**		
Yes	568	97.9
No	12	2.1
**Do you read the labels to obtain nutritional information?**		
Yes	379	65.4
No	201	34.6
**Can you easily understand the terms used in the labels?**		
Yes	284	49.0
No	296	51.0
**Which is the influence of terms such as “light”, “diet”, “enriched” and “source of vitamins”?**		
No influence when buying	130	22.4
Little influence	159	27.4
Average influence	195	33.6
Major influence	96	15.6
**Have you stopped buying any food product because it contained large amounts of calories,** **carbohydrates, fats and/or sodium?**		
Yes	408	70.4
No	172	29.7
**A product with “reduced sodium content” is …?**		
Correct answers	170	29.3
Incorrect answers	410	70.7
**Can a product that claims to have a “low caloric value” be considered light?**		
Correct answers	163	28.1
Incorrect answers	417	71.9
**Can a product that claims to have “low sugar value” be considered light?**		
Correct answers	348	60.0
Incorrect answers	232	40.0

**Table 3:** Factors associated with overweight and obesity in the population from southern Minas Gerais.

	Prevalence (%)	OR	95% CI	*p*-Value
Age				
18–29	39.1	1.000		
30–39	65.2	3.151	2.02–4.97	**<0.001**
40–49	67.9	3.235	1.88–5.63	**<0.001**
50–59	66.2	3.561	1.93–6.72	**<0.001**
Nutritional quality *				
None	66.7	1.000		
Little	65.5	1.031	0.38–2.67	0.951
Important	59.6	0.706	0.27–1.75	0.463
Very much	50.2	0.403	0.15–1.00	0.056
Number of people depending on the income				
1–3	54.5	1.000		
4–8	65.6	1.779	1.17–2.73	0.007
Media that the person usually watches				
Others	78.8	1.000		
Internet	54.9	0.388	0.19–0.74	0.005
Gender				
Male	67.0	1.000		
Female	55.3	0.515	0.32–0.82	0.006
Marital status				
Without a partner	49.3	1.000		
With a partner	62.2	1.403	0.95–2.07	0.087
Sodium **				
Answered incorrectly	56.6	1.000		
Answered correctly	60.1	1.438	0.96–2.17	0.079
Influence ***				
No influence	53.1	1.000		
Little influence	57.9	1.299	0.77–2.18	0.322
Average influence	59.5	1.520	0.92–2.53	0.105
Major influence	59.2	1.629	0.90–2.96	0.106
Flavor ****				
None	61.5	1.000		
Little	52.4	1.071	0.21–5.38	0.933
Important	59.0	1.764	0.46–6.40	0.389
Very much	56.9	2.042	0.54–7.38	0.276
Uses the BHU services				
No	42.5	1.000		
Yes	60.2	2.007	1.22–3.33	**0.006**

* Level of influence of the following terms when buying: [Nutritional quality]. ** A product with “reduced sodium content” is …? *** Influence of the “reduced caloric value”, “light”, “diet”, “enriched” and “source of vitamins” terms. **** Level of influence of the following terms when buying: [Food flavor]. Source: The authors.

## Data Availability

The original contributions presented in this study are included in the article. Further inquiries can be directed to the corresponding authors.

## Supplementary Materials

The following supporting information can be downloaded at: https://www.mdpi.com/article/10.3390/nu18040559/s1. File S1. Data collection questionnaire.
