# Supplementary material for: Nutrition Label Reading and Understanding, Food Advertising Exposure, and Excess Weight Among Brazilian Adults: A Cross-Sectional Study
Source: Nutrients. 2026 Feb 8;18(4):559. doi: 10.3390/nu18040559 (PMC12942836; doi:10.3390/nu18040559)
Supplement: Supplementary file 1 [file nutrients-18-00559-s001.zip › nutrients-4087716-supplementary.pdf]

SUPPLEMENTARY MATERIALS
DATA COLLECTION QUESTIONNAIRE

Email: ____

What is your first name or last name? (For your privacy, you can put only the first letter of your first and last name):

____

Sex:
- ☐ Feminine
- ☐ Masculine

Age: __________

City of residence: _________________________

Education
- ☐ Illiterate
- ☐ Incomplete 1st to 5th grade (formerly 4th grade)
- ☐ Completed grades 1 through 5 (formerly 4th grade)
- ☐ Incomplete 6th to 9th grade (formerly 5th and 8th grade)
- ☐ Completed grades 6 through 9 (formerly 5th and 8th grade)
- ☐ Incomplete 1st to 3rd year of high school
- ☐ completed 1st to 3rd year of high school
- ☐ Incomplete degree
- ☐ Bachelor's degree completed
- ☐ Specialization
- ☐ Master's degree
- ☐ PhD

Marital status:
- ☐ With partner
- ☐ Without a partner

Family income:
- ☐ Up to 1 minimum wage
- ☐ From 1 to 2 minimum wages
- ☐ More than 2 to 3 minimum wages
- ☐ More than 3 to 6 minimum wages
- ☐ More than 6 minimum wages

How many people depend on this income?

Where do you live?

- ☐ Urban area
- ☐ Rural Area

Do you use the services offered at the Basic Health Unit, Family Health Program, or Family Health Strategy (UBS, PSF, and ESF)?

- ☐ Yes
- ☐ No
- ☐ Sometimes

Do you engage in any physical activity? According to the World Health Organization, it is necessary to do 150 minutes of light or moderate physical activity per week (about 20 minutes per day) or at least 75 minutes of more intense physical activity per week (about 10 minutes per day).

- ☐ Yes
- ☐ No

Do you know your current weight? If so, enter your current weight in kilograms here.

Do you know how much you weighed 1 year ago? If so, put it here.

Do you know how tall you are?

Do you know your waist circumference?

Are you the person responsible for grocery shopping in your household?

- ☐ Yes
- ☐ No

Do you usually read food labels?

- ☐ Yes
- ☐ No
- ☐ Sometimes

Do you think nutritional labeling is important?

- [ ] Yes
- [ ] No

Do you use labels to obtain nutritional information and maintain a healthy diet?

- [ ] Yes
- [ ] No

Can you easily understand the terms used on the labels?

- [ ] Yes
- [ ] No

Do you know what "nutritional quality" is?
If possible, describe what you understand about this.

Which of the following media outlets do you most often follow?

- [ ] Television
- [ ] Radio
- [ ] Print media (newspapers, magazines...)
- [ ] Internet

How many hours do you usually spend consuming media per day (including the internet)?

- [ ] Less than 1 hour
- [ ] Between 1 and 3 hours
- [ ] Between 3 and 5 hours
- [ ] More than 5 hours

How much do you trust the media outlets you usually follow?

- [ ] Low confidence
- [ ] Medium confidence
- [ ] A lot of confidence
- [ ] Complete trust

Where do you get information about the nutritional quality of processed foods?

- [ ] Television
- [ ] Radio
- [ ] Print media (newspapers, magazines...)
- [ ] Internet
- [ ] I'm not looking for

Have you ever been drawn to a food product during its advertising and consequently made a purchase?

- [ ] Yes
- [ ] No

How do terms such as "reduced calorie, light, diet, enriched, source of vitamins" found on food labels influence your purchasing decisions?

- [ ] It does not influence the purchase.
- [ ] Small
- [ ] Average
- [ ] Big

Have you ever avoided buying a food because you read on the label that it contains a large amount of calories, carbohydrates, fat, and/or sodium?

- [ ] Yes
- [ ] No

From the following factors, rank from 1 to 4, in ascending order, the one that most influences your food purchases: (1) Not important; (2) Slightly important; (3) Important; (4) Very important.

- [ ] Price
- [ ] Nutritional quality
- [ ] Food flavor
- [ ] Mark
- [ ] Media (advertising on radio, TV, Internet)
- [ ] Practicality
- [ ] Packaging appearance

When you read "reduced energy value" on the label, you can conclude:

- [ ] It's the same as saying that the food has "low energy value".
- [ ] This food provides fewer calories than the traditional food.
- [ ] I am not sure

A food is defined as "reduced in total fat content":

- [ ] The food contains no cholesterol.
- [ ] This food has less fat than its traditional version.
- [ ] I am not sure

When the product label says "cholesterol-free," it means that:

- ☐ The food must not contain cholesterol in its composition under any circumstances.
- ☐ The food may contain a negligible amount of cholesterol or no cholesterol at all.
- ☐ I am not sure

What is a product labeled "reduced sodium"?

- ☐ A food that provides less sodium than traditional food.
- ☐ A food that has undergone a reduction of at least 25% in sodium.
- ☐ I am not sure

Can a product that claims to have a "low calorie count" be considered "light"?

- ☐ Yes
- ☐ No
- ☐ I am not sure

Can a product that claims to have "low sugar content" be considered diet?

- ☐ Yes
- ☐ No
- ☐ I am not sure

How do you think the consumption of these nutrients should be?

	**High**	**Low**	**I am not sure**
**Sugar**	☐	☐	☐
**Saturated and trans fats**	☐	☐	☐
**Sodium**	☐	☐	☐
**Fibers**	☐	☐	☐

Health experts recommend that people eat more, the same amount, or less of the following foods? (Choose one option per food)

	**High**	**Equal**	**Less**	**I am not sure**
**Vegetables**	☐	☐	☐	☐
**Flours**	☐	☐	☐	☐
**Fatty**	☐	☐	☐	☐
**Rich in fiber**	☐	☐	☐	☐
**Fruits**	☐	☐	☐	☐
**Cured meats**	☐	☐	☐	☐

Do you think these foods are high or low in added sugar?

	Rich	Poor	I am not sure
**Banana**	☐	☐	☐
**Natural yogurt**	☐	☐	☐
**Ice cream**	☐	☐	☐
**Refrigerator**	☐	☐	☐
**Kertchup Sauce**	☐	☐	☐
**Dried plums**	☐	☐	☐

Do you think these foods are high or low in fat?

	Rich	Poor	I am not sure
**Pasta (without sauce)**	☐	☐	☐
**Butter**	☐	☐	☐
**Mortadella**	☐	☐	☐
**Nuts**	☐	☐	☐
**Cottage cheese**	☐	☐	☐
**Margarine**	☐	☐	☐

Do you think these foods are high or low in salt?

	Rich	Poor	I am not sure
**Sausage**	☐	☐	☐
**Mass**	☐	☐	☐
**Canned tuna**	☐	☐	☐
**Red meat**	☐	☐	☐
**Mixed vegetables**	☐	☐	☐
**Plate cheese**	☐	☐	☐

Do you think these foods are high or low in fiber?

	Rich	Poor	I am not sure
**Wheat bran**	☐	☐	☐
**Bananas**	☐	☐	☐
**Chard**	☐	☐	☐
**Red meat**	☐	☐	☐
**Broccoli**	☐	☐	☐
**Potatoes with skins on, cooked**	☐	☐	☐

Do you consider these fatty foods to be high or low in saturated fat?

	Rich	Poor	I am not sure
**Sardine**	☐	☐	☐
**Whole milk**	☐	☐	☐
**Oil**	☐	☐	☐
**Red meat**	☐	☐	☐
**Margarine**	☐	☐	☐
**Chocolate**	☐	☐	☐
